# Ophthalmology

**Published:** 1896-03

**Authors:** Alvin A. Hubbell

**Affiliations:** Buffalo, N. Y.; Professor of ophthalmology and otology in the Medical Department of Niagara University


### Progress in Medical Science.

### OPHTHALMOLOGY.

CONDUCTED BY ALVIN A. HUBBELL, M. D., Buffalo, N. Y.
Professor of ophthalmology and otology in the Medical Department of Niagara University.

### FORMALIN AS A PRESERVING AND HARDENING AGENT FOR EYE SPECIMENS.

DR. WILLIAM H. WILDER, of Chicago, (*Ophthalmic Record*, October, 1895,) writes that there are many disadvantages accompanying the use of Müller's fluid and alcohol for hardening eye specimens. The former so discolors the tissues that beautiful microscopic preparations are difficult to obtain; while the latter, unless cautiously used, causes much shrinking and distortion of the specimens.

These objections, he holds, may be overcome by the use of formalin, a 40 per cent. aqueous solution of formaldehyde. This may be diluted with water to any desired strength. The author has found a 5 per cent. to be the most useful.

Immediately after enucleation the blood is washed from the eyeball and it is dropped into a bottle containing several ounces of a 5 per cent. solution. In a few days it will be hard enough to bisect, which can be done with a razor, without first freezing, provided the contents of the globe are solid; if, however, the vitreus, either normal or fluid, be present, it is best to freeze the eyeball before cutting, as the formalin does not harden the vitreus sufficiently.

The half to be preserved is then treated according to the method of Priestly Smith, by placing it for twenty-four hours in a 33⅓ per cent. and then a 50 per cent. solution of glycerine and water.

After this it is mounted in a glass cup in glycerine jelly.

---

### WHICH CANALICULUS TO SLIT IN PROBING THE NASAL DUCT.

MR. J. B. STORY, (Dublin,) writing on probing the nasal duct, in the *Ophthalmic Review*, June, 1895, says: "The obstruction to be overcome is situated in the nasal duct, and in order to pass the large-sized probes, which are now and have been for many years so extensively employed in this country and in America, it is necessary to divide one of the canaliculi. It might seem a matter of no importance whether the upper or the lower canaliculus is selected to suffer the necessary mutilation, but the choice is not a matter of indifference; the results are far better and more easily attained by selecting the upper instead of the lower. . . . . My own practice in this matter dates from many years back, and was adopted in consequence of my observing many patients who had been treated by division of the lower canaliculi, whose nasal ducts were perfectly patent, but who, nevertheless, still suffered from constant epiphora, and whose watery eyes were observable to the most casual observer. Since I have divided the upper canaliculus, I may have failed in some cases to obtain a permanent patency of the nasal duct, but I have never failed to relieve the epiphora in the cases in which I succeeded in curing the stricture of the duct."

---

### ASTIGMIA VERSUS ASTIGMATISM.

GEORGES MARTIN, (*Annales d' Oculistique*, March, 1895,) in a brief note proposes to substitute the word astigmia for astigmatism, as being, first, shorter and therefore more convenient, and secondly—a more important reason—etymologically correct. From the point of view of accurate derivation there is, we believe, no doubt that he has reason on his side.

---

### THE RADICAL OPERATIVE TREATMENT OF TRICHIASIS.

Mr. KENNETH SCOTT, of Cairo, (*The Ophthalmic Review*, September, 1895,) describes a method of operation which he now employs and has termed, for the want of a better word to express its nature, "tarsal reposition." Having performed it on a large number of cases with almost invariable success, he now feels justified in making the matter generally known.

The instruments requisite for operation are the following: (1) A scalpel, or a Beer's cataract knife, with a well-rounded off and sharp-cutting point. (2) An ordinary entropian spatula, which is more convenient to use if a narrow square metal ridge is added across the back, enabling the operator to hold it in position, when necessary, with the fifth finger of the left hand. (3) A pair of strong double-bladed fixation forceps, closed by a sliding catch. (4) Needle-holding forceps, two small double-eyed curved needles for wire, and very fine silver wire, thoroughly softened before use by being passed through the flame of a spirit lamp.

The operation may be described as follows: the eyebrow and both surfaces of the eyelids having been thoroughly cleansed, the spatula is passed into the conjunctival fornix, under the upper eyelid, which is then everted; an incision is made on this conjunctival surface of eyelid, parallel to, and at a distance of about 2 m.m. from the margin; it must divide the tarsus completely in its whole thickness, and from end to end. Any bleeding which occurs is easily arrested by pressure. The eyelid is replaced in position, and its divided margin grasped between the blades of the forceps; the catch is closed, and the handle of the forceps then carried upward, so as to rest against the eyebrow, and thus forcibly evert the separated portion of the tarsus which carries the eyelashes. One needle armed with silver wire, about 18 inches (45 c. m.) long, is sufficient for each eyelid; it is passed vertically downward, through the skin-surface of the center of the lid into the substance of the upper portion of tarsus and emerging in the middle of its divided edge, then enters the original anterior surface of the lower separated and everted portion of tarsus, to be finally brought out on the free margin of the eyelid, midway between the eyelashes and conjunctival edge. Two other sutures are similarly introduced, one toward each end of the eyelid. The opposing ends of these three sutures, which should be left long, are now separately twisted together, not so tightly as to cause constriction, but just firmly enough to retain the eyelid margin in its everted position. The forceps are removed. The remaining part of the suture is now passed along in the tissue of the eyebrow, from one extremity to the center, at which points the corresponding twisted strands of wire are attached to it; the needle is re-introduced at the center of the eyebrow, close to its point of emergence, and is brought out at the opposite end, where the third remaining twisted suture is secured to it. When the lower eyelid is the site of operation, the tissue of the cheek is used as a fixation point, instead of the eyebrow.

The operation occupies only about three and one-half to four minutes. Usually a requisite amount of anesthesia can be produced by the simple injection of cocaine in the eye, as the operation is such a rapid one. Subcutaneous injections of cocaine in these cases has hitherto been found unsatisfactory. To several patients who were very nervous, and sometimes also to women, either nitrous-oxide gas or chloroform has had to be administered, but, as a rule, the patients have borne the operation very well.

No dressing other than a dusting of dermatol, etc., in powder, need be employed; the parts should be kept absolutely clean, and the stitches removed on the fifth to seventh day. This is best done by first dividing the three twisted strands, then the eyebrow sutures on each side of and close to the central knot should be divided and the two halves from either end withdrawn ; then each loop embracing the lid margin is cut, and the wire being soft, is easily withdrawn by simple traction.

After the lapse of four to ten days from the removal of the stitches, it is most difficult to realise, except on very close examination, that the eyelid has ever been interfered with.

